# Deploying a metabolic dysfunction-associated steatohepatitis consensus care pathway: findings from an educational pilot in three health systems

**DOI:** 10.1186/s12875-024-02517-y

**Published:** 2024-07-20

**Authors:** Sonal Kumar, Arpan Mohanty, Parvez Mantry, Robert E. Schwartz, Madeleine Haff, George Therapondos, Mazen Noureddin, Douglas Dieterich, Nigel Girgrah, Kristi Cohn, Mohanish Savanth, Michael Fuchs

**Affiliations:** 1 Weill Cornell Medicine, 1305 York Avenue, 4 Floor, New York, NY 10021 USA; 2 Boston Medical Center, 85 E. Concord Street, 7 Floor, Boston, MA 02118 USA; 3 Methodist Health System, 1411 N Beckley Ave #268, Dallas, TX 75203 USA; 4 Ochsner MultiOrgan Transplant Institute at Ochsner Health, 1514 Jefferson Hwy, New Orleans, LA 70121 USA; 5 Houston Research Institute, Houston Methodist Hospital, 1155 Dairy Ashford Rd, Suite 200, Houston, TX 77079 USA; 6 Icahn School of Medicine at Mount Sinai Health System, 1 Gustave L. Levy Place, New York, NY 10029-5674 USA; 7 NASHNET, 29 Broadway, Floor 26, New York, NY 10003 USA; 8 Central Virginia VA Health Care System, 1201 Broad Rock Blvd, Richmond, VA 23249 USA; 9 Virginia Commonwealth University, 1200 E Broad Street, West Hospital, 14 Floor, Box 980341, Richmond, VA 23298-0341 USA

**Keywords:** Metabolic dysfunction-associated steatotic liver disease, Metabolic dysfunction-associated steatohepatitis, MASLD, MASH, Care pathway, Guidelines-based care, Provider education, Non-invasive diagnostics

## Abstract

**Background:**

Metabolic dysfunction-associated steatotic liver disease (MASLD), formerly referred to as nonalcoholic fatty liver disease, impacts 30% of the global population. This educational pilot focused on the role primary care providers may play in the delivery of guidelines-based metabolic dysfunction-associated steatohepatitis (MASH) care.

**Objective:**

Accelerate the application of guidelines-based MASH care pathways to clinical workflows.

**Methods:**

A panel of six hepatologists was convened in 2021 to develop the care pathway and the subsequent pilot occurred between 2022 – 2023. The pilot was conducted across three U.S. health systems: Boston Medical Center (Boston), Methodist Health System (Dallas), and Weill Cornell Medicine (New York). Clinicians were educated on the care pathway and completed baseline/follow-up assessments. 19 primary care clinicians participated in the educational pilot baseline assessment, nine primary care clinicians completed the two-month assessment, and 15 primary care clinicians completed the four-month assessment. The primary endpoint was to assess clinician-reported adherence to and satisfaction with the care pathway. The pilot was deemed exempt by the Western Consensus Group Institutional Review Board.

**Results:**

At baseline, 38.10% (*n* = 8) of respondents felt they had received sufficient training on when to refer a patient suspected of metabolic dysfunction-associated liver disease to hepatology, and 42.86% (*n* = 9) had not referred any patients suspected of metabolic dysfunction-associated liver disease to hepatology within a month. At four months post-intervention, 79% (*n* = 15) of respondents agreed or strongly agreed they received sufficient training on when to refer a patient suspected of metabolic dysfunction-associated liver disease to hepatology, and there was a 25.7% increase in self-reported adherence to the institution’s referral guidelines. Barriers to care pathway adherence included burden of manually calculating fibrosis-4 scores and difficulty ordering non-invasive diagnostics.

**Conclusions:**

With therapeutics anticipated to enter the market this year, health systems leadership must consider opportunities to streamline the identification, referral, and management of patients with metabolic dysfunction-associated steatohepatitis. Electronic integration of metabolic dysfunction-associated steatohepatitis care pathways may address implementation challenges.

## Background

Metabolic-dysfunction associated steatotic liver disease (MASLD), formerly known as nonalcoholic fatty liver disease (NAFLD), affects approximately 30% of the global population and is defined as the presence of hepatic steatosis in conjunction with at least one cardiometabolic risk factor and no other discernible cause [1, 2]. An estimated 20% of MASLD cases progress to the more severe form of the disease called metabolic dysfunction-associated steatohepatitis (MASH), formerly known as nonalcoholic steatohepatitis (NASH) [3]. MASH is characterized by an accumulation of fat within the liver, which may cause liver inflammation, stiffness, or damage. Left untreated and unmanaged, over the course of many years MASH can lead to poor long-term outcomes including cirrhosis, hepatocellular carcinoma (HCC), liver transplantation, or death [4].

Common cardiometabolic risk factors for MASLD and MASH include obesity, type 2 diabetes, insulin resistance, hypertension and hyperlipidemia which can potentially contribute to increased complications [5]. Approximately 50 to 70% of people with diabetes are believed to have MASLD [6]. MASLD/MASH guidelines from several different organizations, such as the American Association for the Study of Liver Disease (AASLD), the American Diabetes Association (ADA), the American Gastroenterological Association (AGA), and the American Association of Clinical Endocrinology (AACE), have been published within the last five years with a focus on screening high-risk populations [7–10]. However, MASLD/MASH care pathways developed by the AGA and AACE do not account for variances in patient populations, test availability, or real-world limitations (e.g., access to diagnostics).

Despite the increasing prevalence of disease and range of commonly seen risk factors, gaps in primary care practitioner (PCP) awareness around MASH care still exist. A previous study identified lack of confidence in diagnosing MASLD and inconsistent approaches to disease management as barriers for PCPs [11]. Additionally, guidelines do not provide substantial direction on disease management or specific treatment for MASLD outside of lifestyle modifications. Weight loss is a key part of management, and options can include improving diet and exercise habits, as well as therapeutic and bariatric/metabolic surgical interventions. For many patients with elevated BMIs, weight loss of 3–5% can reduce steatosis, 7% weight loss can result in MASH regression, and 10% weight loss can lead to fibrosis regression [12].

The recent Federal Drug Administration (FDA) approval of Rezdiffra™ (resmetirom) has reinvigorated conversations amongst stakeholders across the care continuum who are eager to identify opportunities to proactively diagnose, risk stratify, and refer patients at-risk of progression to inflammation and fibrosis [13]. It is essential that MASH care pathways are implemented now to accelerate the adoption of guidelines and provide patients with access to appropriate treatment options earlier in their disease progression, when prevention of negative long-term outcomes is possible.

This study aimed to evaluate PCP awareness of existing MASLD care protocols and assess feasibility of real-world implementation of a consensus MASH care pathway within three U.S. health systems. Learnings from this paper could be used to consider future opportunities to optimize MASH care pathway implementation across various care delivery environments and encourage adoption of guidelines-based clinical care workflows. While the care pathway implementation pilot was deployed prior to the nomenclature changes presented by multinational liver societies in June 2023, the foundational structure of the pathway remains unchanged and has been updated to maintain relevance over time as guidelines (e.g., AACE, AASLD) continue to evolve. Additionally, 99% of clinical indicators for NAFLD meet MASLD criteria and it was determined that these terms can be used interchangeably [14].

## Methods

### Consensus care pathway development

In November 2021, an expert panel of six hepatologists convened to develop a consensus care pathway for MASLD/MASH based upon guidelines and literature available at the time. The expert panel included hepatologists from leading health systems representing over approximately 6.9 million lives across the United States including Cedars Sinai Medical Center (Los Angeles, CA), the Central Virginia VA (Richmond, VA), Methodist Health System (Dallas, TX), Mount Sinai Health System (New York, NY), and Ochsner Health (New Orleans, LA). The panel reviewed pre-existing literature, including AASLD and AGA guidelines available at the time, and convened during a 2-h hybrid meeting to discuss challenges and gaps within MASH care delivery, optimal state care pathways, and the role of biomarkers in future MASH care pathways. Following the roundtable meeting, a draft pathway was developed for panel review. Panel feedback was gathered via ad hoc calls and email correspondence, and over the course of 2–3 months the pathway was refined until all panel participants confirmed consensus with the pathway to be piloted. The pathway was then reviewed post pilot implementation by the panel for relevancy given evolving U.S. guidelines and implementation considerations captured.

### Implementation pilot design

An educational intervention to implement this pathway was piloted across three health systems from 2022 – 2023: Boston Medical Center (Boston, MA), Methodist Health System (Dallas, TX), and Weill Cornell Medicine (New York, NY). These three institutions were selected for their geographic diversity, differences in patient populations served, varying levels of access to non-invasive diagnostics, and PCP willingness to engage. Prior to deploying the intervention, a study protocol was jointly developed by these sites and reviewed by their respective research offices, before receiving an exemption from WCG-IRB.

### Settings & participants

The intervention involved the respective principal investigators (PI) conducting an educational session for five to nine primary care practitioners (PCPs) at each pilot site selected via convenience sampling. PCPs were defined as clinicians (MDs, DOs), nurse practitioners (APRN-NP, FNP, ANP, CNP) or physician assistants (PA, PA-C) who were licensed to provide primary care services to patients. Each system conducted one educational session either in-person or virtually (depending upon system preference & COVID guidelines).

### Intervention

Education was presented utilizing a standardized 30-slide education deck, followed by a Q&A session. Educational content included background information on MASLD/MASH burden of disease & common comorbidities, disease staging information, common clinical characteristics, guideline recommendations, the consensus care pathway overview, and information on each of the non-invasive diagnostics included within the pathway. Educational content was developed based upon Investigator input on potential educational gaps for PCPs given past experience or frequently asked questions.

### Data collection & analysis

Provider surveys were jointly created by the Investigators and NASHNET staff with the intent to capture qualitative and quantitative data relating to provider awareness of the disease, guidelines-recommended use of non-invasive tests (NITs), and self-reported process measures relating to the care pathway adoption. Survey questions were crafted leveraging a mix of Likert scale, ranking, and open response question types and validated by Investigators. The surveys were deployed at baseline, 2-months post intervention, and 4-months post intervention to evaluate PCP understanding of MASLD/MASH prevalence, risk stratification and referral criteria, appropriate use of non-invasive diagnostics, and adherence to the consensus care pathway over time. Responses were captured via an electronic survey platform. No further proactive education was provided to pilot participants, but participants were encouraged to reach out to the Investigators/their fellow hepatologists with relevant questions. Table 1 illustrates the baseline and follow-up survey questions. Of note, at the time of pilot implementation, the MASLD/MASH nomenclature was not yet widely accepted. While the survey assessments were deployed using the NAFLD/NASH nomenclature, discussion of results will utilize MASLD/MASH nomenclature.

**Table 1:** Baseline & follow up survey questions

Survey question	Question type	Survey cadence
1. Please indicate your mother’s first, middle, and last name initials below. (Note: your survey responses will be kept anonymous. We ask for this information to track baseline v. follow up results)	Open-ended text box	Baseline 2-month follow up 4-month follow up
2. Please indicate your institution below:	Multiple choice	Baseline 2-month follow up 4-month follow up
3. How much do you agree or disagree with the following statements? a. I believe that NAFLD is a highly prevalent disease b. I understand the risk factors for developing NAFLD c. I understand the difference between NAFLD and NASH d. I know how to counsel a patient regarding NAFLD e. I have received sufficient training on when to refer a patient suspected of NAFLD/NASH to hepatology f. I have received sufficient training on the appropriate use of non-invasive tests (NITs) to detect NALFD/NASH and inform referral decisions	Likert scale (Strongly disagree, disagree, neutral, agree, strongly agree)	Baseline 2-month follow up 4-month follow up
4. Does your institution have a formal referral protocol for patients suspected of NAFLD/NASH to hepatology?	Multiple choice	Baseline 2-month follow up 4-month follow up
5. In the past 12 months have you performed any of the following: a. Used the FIB-4 score to risk stratify patients suspected of NAFLD/NASH b. Referred a patient suspected of NAFLD/NASH to hepatology c. Ordered any other non-invasive test (apart from FIB-4) to detect NAFLD/NASH? Examples include ELF^a^, FibroScan, & MRE i. If yes, which non-invasive test(s)?	Multiple choice (yes/no)	Baseline 2-month follow up 4-month follow up
6. How much do you agree or disagree with the following? a. I am familiar with my organization’s protocol for screening and identifying patients at risk for NAFLD/NASH b. I follow my organization’s protocol for screening and identification of patients at risk for NAFLD/NASH the majority of the time c. My organization’s protocol for screening and identification of patients at risk for NAFLD/NASH is aligned with evidence-based best practice?	Likert scale (Strongly disagree, disagree, neutral, agree, strongly agree)	Baseline 2-month follow up 4-month follow up
7. When screening patients for NAFLD/NASH, in which order do you use the following modalities?	Rank order: liver function tests, FIB-4, ELF, VCTE, referral to hepatology	Baseline 2-month follow up 4-month follow up
8. In the past month, approximately what percent of your patients did you refer to hepatology for suspected NAFLD/NASH?	Open-ended text box	Baseline 2-month follow up 4-month follow up
9. What factors, if any, might cause you to change the order of the modalities you use in screening patients for NAFLD/NASH? For example, a patient’s insurance coverage or test availability	Open-ended text box	Baseline 2-month follow up 4-month follow up
10. How many years have you been in practice?	Multiple choice	Baseline
11. What is your position?	Multiple choice	Baseline
12. What is your primary specialty?	Multiple choice	Baseline
13. How do you spend the majority of your time?	Multiple choice	Baseline
14. Do you see patients at a Faculty Practice site or Resident Teaching site?	Multiple choice	Baseline
15. How would you rate ease of adoption of your organization’s protocol for screening and identifying patients with NAFLD/NASH (with 0 being very difficult and 10 being very easy)?	Scale (1 – 10)	2-month follow up 4-month follow up
16. Please describe your answer to the above	Open-ended text box	2-month follow up 4-month follow up
17. Have you faced any barriers to implementing your organization’s protocol for screening and identifying patients with NAFLD/NASH? Please describe	Open-ended text box	2-month follow up 4-month follow up
18. What resources/tools would help you in your adoption of the organization’s protocol for screening and identifying patients with NAFLD/NASH?	Open-ended text box	2-month follow up 4-month follow up

Survey questions were created prior to the nomenclature change in 2023 and are referenced in portions of this report using the previous nomenclature

^a^ELF Score: Low Risk < 9.80; Mid Risk ≥ 9.80 to < 11.30; High Risk ≥ 11.30

## Results

### Adapting guidelines to real-world settings

**Fig. 1:**
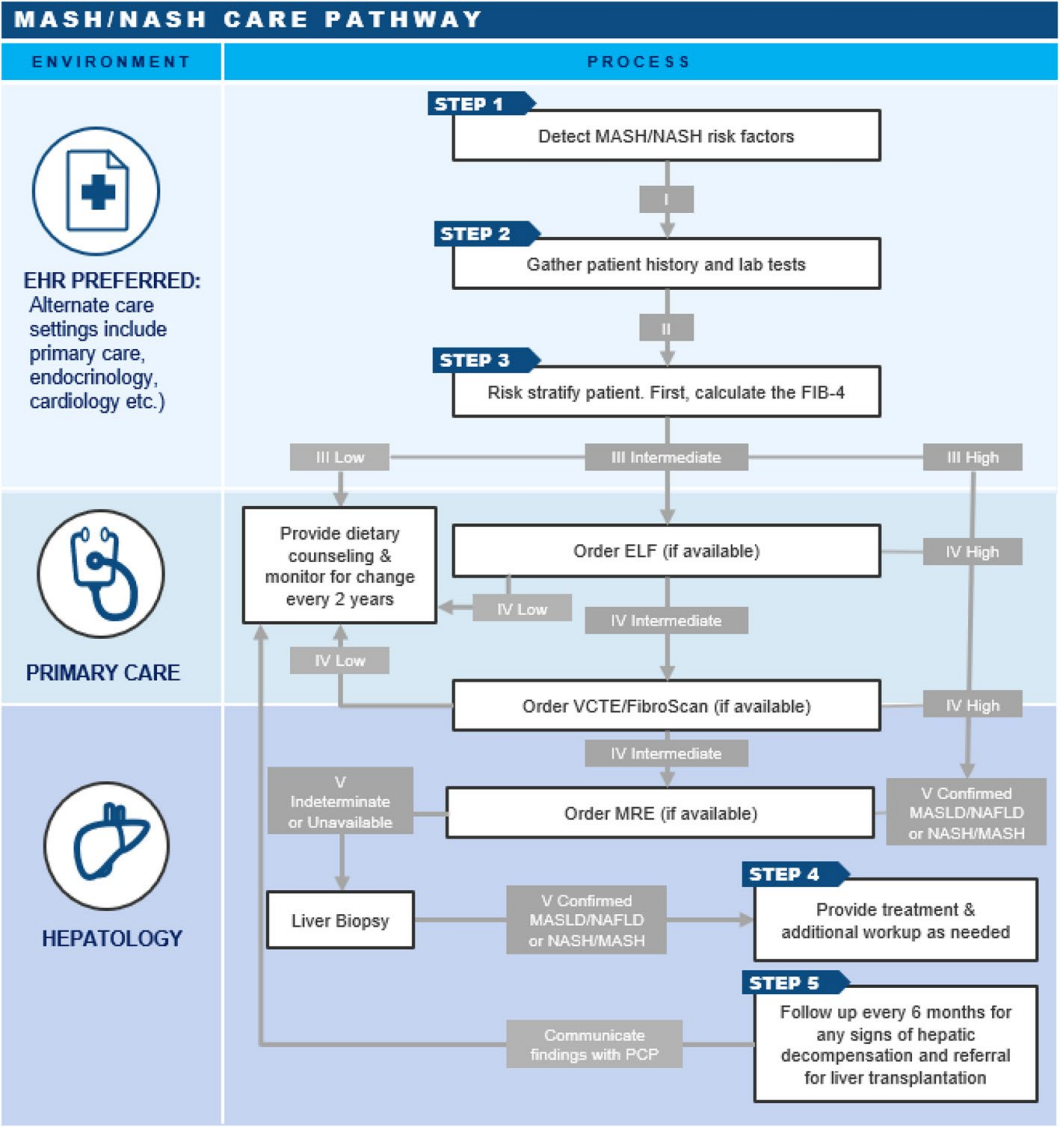
Consensus MASH care pathway**. ***FIB-4:* < *1.3 (low), 1.3 – 2.67 indeterminate,* > *2.67 high; ELF:* < *9.8 (low),* ≥ *9.8 (high).* Note: Care pathway above was developed and disseminated prior to the nomenclature change in 2023

The expert panel of hepatologists defined the following consensus MASH care pathway (Fig. 1) as well as the associated decision criteria and implementation considerations (Table 2). The care pathway has been updated to account for the updated MASLD/MASH nomenclature. The panel recommended refinements to the AGA care pathway, including stepwise utilization of FIB-4 followed by ELF (if available) followed by VCTE (if available). Utilization of a second-tier blood-based diagnostic within the primary care environment was recommended to decrease the number of indeterminate patients recommended for referral to VCTE, which is primarily available within hepatology practice settings.

**Table 2:** Pathway Decision Node Criteria & Implementation Considerations

Process Step	Decision Criteria Definition	Implementation Considerations
i	Proceed if ≥ 1 of the following criteria apply: • Evidence of fatty liver • BMI ≥ 30* • Type 2 diabetes • Elevated liver test • Hyperlipidemia • Hypercholesterolemia • Hypertension • Renal Disease	• Recommend automating within electronic health record (EHR) o *Data source: problem list, discrete data fields, screening questions*
ii	Proceed after assessing following: • CBC and liver function tests • Patient history (e.g., alcohol use, medications) • No other documented cause of liver disease • Not already followed in hepatology	• Recommend automating within EHR • Historic CBC or liver function tests can be used if results gathered in past 6 months, otherwise new labs will need to be ordered • Other documented causes of liver disease may include: HBV, HCV, biliary obstruction, HCC, Wilson disease, Budd Chiari syndrome, or alcoholic liver disease (e.g., consumption of > 14 drinks/week for women or 21 drinks/week for men) • Patient history can be sent out via patient portal questionnaire or via phone by population health coordinator/case manager
iii	FIB-4 cut off values: • High: > 2.67 • Indeterminate: 1.3 – 2.67 • Low: < 1.3	• Recommend automating within EHR o Auto-refer patients with FIB-4 > 2.67 o Gain PCP buy in to flag indeterminate patients for additional workup • FIB-4 interpretation can also be completed by a population health coordinator/case manager
iv	ELF cut off values: • High: ≥ 9.8 • Low: < 9.8 FibroScan cut off values: • High: < 15 kPa • Indeterminate: 8 ≤ kPa ≤ 15 • Low: < 8 kPa	Usage of ELF versus FibroScan will depend on factors, including: • Access to FibroScan (e.g., if no access to FibroScan within surrounding area, shipping out a blood sample may lessen burden for patient, so ELF is preferred) • Payer coverage of ELF (e.g., if ELF coverage is limited, then FibroScan may be preferred)
v	Confirmed NAFLD or NASH: • Evidence of NAFLD or NASH in imaging or biopsy Indeterminate or Unavailable: • MRE results were found inconclusive or clinician does not have access to MRE	Treatment dependent upon fibrosis score: • 0–1: ILI Counselling (diet, exercise, weight loss management, dietician counselling) and diabetes control (if applicable) • 1 < F < 3: All of Above + Clinical Trial (if available & patient consents) + Annual Fibrosis Assessment + Annual Cardiovascular Risk Assessment • 3 ≤ : All of Above + Clinical Trial (if available & patient consents) + HCC Screening and Variceal Assessment + Follow up Every 6-Months for Signs of Hepatic Decompensation & Referral for Liver Transplantation

*Health systems may consider further stratifying certain populations (e.g., consider lower BMI > 18.5 for Asian Americans to account for lean MASH). Note: Care pathway criteria above were developed and disseminated prior to the nomenclature change in 2023

The consensus care pathway was then deployed across three pilot sites. Twenty-one providers participated in the baseline assessment, including five participants from BMC, nine participants from Methodist Health System, and seven participants from Weill Cornell Medicine. Two baseline surveys were excluded from the analysis, as they were gastroenterology fellows and not the primary focus of the intervention. Eighteen respondents were primary care physicians and one respondent was a primary care nurse practitioner. Of the 19 eligible respondents, 21% have been practicing for < 5 years, 42% have been practicing for 5–20 years, and 37% have been practicing for > 20 years. The follow-up surveys administered 2 months and 4 months after the initial intervention were completed by 9 and 15 eligible participants, respectively.

### Awareness of existing protocols

While all respondents at baseline agreed or strongly agreed that MASLD is a highly prevalent disease, only 32% (*n* = 6) felt they had received sufficient training on when to refer to hepatology, and 37% (*n* = 7) felt they had received sufficient training on the appropriate use of non-invasive tests (NITs) to inform MASLD/MASH referral decisions.

79% (*n* = 15) were unsure or did not believe their institution had a formal referral protocol for patients suspected of MASLD/MASH. Within the past month, 47% (*n* = 9) had not referred any patients suspected of MASLD/MASH to hepatology, 37% (*n* = 7) indicated they referred between 1–10% of their patients to hepatology for suspected MASLD/MASH, and 16% (*n* = 3) indicated they referred > 10% of their patients to hepatology for suspected MASLD/MASH.

**Fig. 2:**
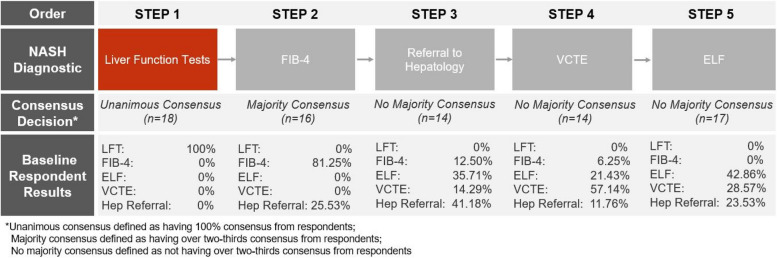
Order of MASH diagnostics in referral pathway by baseline responses. ELF: Enhanced Liver Fibrosis; FIB-4: Fibrosis-4; VCTE: Vibration-controlled transient elastography

While there was consensus among participants that liver enzymes were the first step to determining if a patient was at risk of MASLD/MASH, there was a lack of consensus amongst participants regarding the next step of the referral protocol, with 81% believing that an elevated FIB-4 (> 1.3) would be the next step, and 24% believing referral to hepatology would be the next step. The full breakdown of the baseline responses is included in Fig. 2.

Both the 2- and 4-month surveys reaffirmed that 100% of participants who completed the follow up surveys agree or strongly agree that MASLD is a highly prevalent disease. From baseline to 4-months, the percent of participants who either agreed or strongly agreed that they had received sufficient training on when to refer a patient suspected of MASLD/MASH to hepatology increased from 32 to 80%, with zero participants indicating they disagreed or strongly disagreed with the statement.

From baseline to 4-month follow up results, there was a 26% increase in self-reported adherence to the institution’s referral guidelines, and in the same time frame, the proportion of participants who reported calculating a FIB-4 score within the past 12-months rose from 53 to 73%. Results are summarized in Table 3.

**Table 3:** Summary of baseline and 4-month follow up participant responses

Question:	Baseline responses	4-month Follow up responses
How much do you agree or disagree with the following statement? I have received sufficient training on when to refer a patient suspected of NAFLD/NASH to hepatology	Strongly Agree (*n* = 1) Agree (*n* = 5) Neutral (*n* = 6) Disagree (*n* = 7) Strongly Disagree (*n* = 0)	Strongly Agree (*n* = 5) Agree (*n* = 7) Neutral (*n* = 3) Disagree (*n* = 0) Strongly Disagree (*n* = 0)
How much do you agree or disagree with the following? I follow my organization’s protocol for screening and identification of patients at risk for NAFLD/NASH the majority of the time	Strongly Agree (*n* = 1) Agree (*n* = 2) Neutral (*n* = 4) Disagree (*n* = 9) Strongly Disagree (*n* = 3)	Strongly Agree (*n* = 2) Agree (*n* = 5) Neutral (*n* = 6) Disagree (*n* = 2) Strongly Disagree (*n* = 0)
In the past 12 months have you performed any of the following: Used the FIB-4 score to risk stratify patients suspected of NAFLD/NASH?	Yes (*n* = 10) No (*n* = 9)	Yes (*n* = 11) No (*n* = 4)

### Factors impacting care pathway adoption

At baseline, participants noted that factors that may cause them to change the order of modalities used in screening patients for MASLD/MASH included: test availability (*n* = 7), further provider education/understanding of tests (*n* = 3), patient motivation/interest in further workup (*n* = 2), test coverage/cost (*n* = 2), ease of interpretation (*n* = 1), and access to studies (*n* = 1). One participant did not select any factors (*n* = 1).

Since the pilot institutions did not have pre-existing policies or formalized care pathways in place, follow-up feedback from participants regarding their experience with pathway implementation and factors impacting adoption was captured at the 2- and 4-month intervals in Table 4.

**Table 4:** Summary of aggregated follow-up respondent feedback on care pathway implementation

Questions	Comments/feedback from providers
What factors, if any, might cause you to change the order of the modalities you use in screening patients for NAFLD/NASH? (for example, patient’s insurance coverage or test availability)? [Open response]	Top 4 factors: 1. Test availability: 42% (*n* = 18) 2. Provider education/awareness: 19% (*n* = 8) 3. Insurance coverage: 168% (*n* = 7) 4. Patient interest: 7% (*n* = 3)
Please rate ease of adoption of your organization’s protocol for screening and identifying patients with NAFLD/NASH (with 0 being very difficult and 10 being very easy)	Average Score of 2-month survey: **4.6** Average Score of 4-month survey: **6.5**
	Comments key themes: • Difficulty remembering screening guidelines and protocol during busy PCP workflow (*n* = 8) • Unclear instructions to order and interpret FibroScan (*n* = 3) • Added time to calculate FIB-4 scores (*n* = 5)
Have you faced any barriers to implementing your organization’s protocol for screening and identifying patients with NAFLD/NASH? Please describe. [Open response]	• Limited FibroScan availability (*n* = 5) • Lack of automation and Epic prompts regarding FIB-4 and overall protocol (*n* = 7) • Unclear insurance coverage (*n* = 1) • Lack of PCP awareness around protocol (*n* = 4)
What resources/tools would help you in your adoption of the organization’s protocol for screening and identifying patients with NAFLD/NASH? [Open response]	• Integrated Epic prompts (e.g., drop down order menu, automated FIB-4 calculator, visible algorithm) (*n* = 13) • Increased access to hepatology and FibroScan (*n* = 4) • Easily accessible protocol (either on institutional website or within Epic) (*n* = 4)

Survey questions above were developed and administered prior to the nomenclature change in 2023

## Discussion

The baseline survey, distributed pre-education, highlighted a significant gap between understanding disease prevalence and hepatology referrals. Although participating PCPs recognize the high prevalence of MASLD, there remains a gap in translating this awareness into referrals to hepatology. A possible contributing factor to this finding is the limited proportion of participants who felt they had received sufficient training regarding appropriate referral practices for a patient suspected of MASLD/MASH. The gap was further reinforced by PCPs indicating a need for increased education and awareness regarding tools available to them to self-manage MASLD/MASH patients in primary care, risk stratify, and refer to hepatology as needed. Spann, et al. also highlight a lack of non-invasive screening availability within some primary care populations, which can hinder appropriate referrals [15].

Follow-up assessments after the intervention indicate that while education and application of the MASH care pathway to clinical workflows led to an increase in the number of referrals, barriers to implementation persist. Provider understanding of the high prevalence of MASLD remained steady, and confidence in their NIT utilization and hepatology referral improved. Participants listed barriers to adoption, including overall unfamiliarity regarding the protocol, excessive time associated with manually calculating a FIB-4 score, difficulty ordering non-invasive tests (e.g., FibroScan) within the electronic health record (EHR), and unclear insurance coverage. While insurance coverage of non-invasive diagnostics is likely to evolve once MASH therapeutics become available, respondent feedback indicates further action is needed across the clinical community to integrate tools to enable adoption of MASH care pathways within electronic workflows. Building out clinical decision support (CDS) enabled tools may facilitate the accelerated adoption of MASH care pathways. These tools could include auto-generating FIB-4 scores for patients with risk factors indicated in their problem lists, establishing a FIB-4 order set, considering reflexive test ordering, and/or creating a checklist within the EHR for clinicians to easily order second-line diagnostics or refer to hepatology. Continued research is needed to generate evidence demonstrating the impact of MASLD/MASH care pathways on long-term clinical outcomes as well as adoption across a broader PCP community.

The MASLD consensus care pathway was developed recognizing a need to accelerate the timeline between the release of guidelines and application of evidence-based care to health system workflows. The pathway was built upon the foundation set by the AGA care pathway and refined as additional U.S. professional associations (e.g., AACE, AASLD) released updated guidelines over time. The expert panel discussion illuminated many challenges and opportunities relating to the screening, identification, stratification, and management of NAFLD/NASH, including:

Challenges:

- Lack of clear screening and referral guidelines
- Limited insurance coverage of non-invasive diagnostics
- Lack of buy-in outside of the hepatology community (e.g., administration) regarding the importance of improved metabolic care
- Possibility of patients opting out of liver biopsy where recommended, despite being gold standard for diagnosis
- Heterogeneity of populations, diagnostic availability, variability in testing results.

Opportunities:

- Multidisciplinary clinician engagement
- NIT strategy alignment
- Care pathway integration into electronic workflows
- Evidence generation supporting implementation of care pathways

Expert panelists highlighted the importance of ensuring the care pathway is simple but provides some flexibility for screening and diagnosis to account for regional access to diagnostics and variances in patient population and care delivery archetype. Previous studies have highlighted the need for and efficacy of risk stratifying patients in primary care by deploying NITs, especially FIB-4, in a multi-tiered approach to streamline referrals to hepatology [16–18]. Recognizing the importance of delaying disease progression, the consensus care pathway is intended to help PCPs understand the role they can play in MASH care delivery. However, this paper is one of the first to assess a proposed MASH care pathway with a focus on PCPs and demonstrates how provider education is a key part of a multi-strategy pathway.

With therapeutics anticipated to be available within the U.S. in 2024, this presents an opportunity to raise awareness of MASLD/MASH, improve early detection and diagnosis, and link patients to appropriate care, whether it be continued management in primary care, lifestyle intervention, referral to hepatology, therapeutic intervention (once available), or a combination.

Limitations to this pilot initiative included the participant sample size and follow-up data gathered. While the Investigators anticipate the educational intervention will translate to larger group learning settings (e.g., grand rounds), this intervention was conducted within smaller group settings. Thus, there is potential bias introduced from the participant sample size, as primarily providers with an existing interest in steatotic liver disease may have chosen to participate. Additionally, this pilot focused on a survey follow-up period of 4 months. To demonstrate long-term knowledge retention beyond that, additional investigation would be required. Another limitation to acknowledge is that no objective data collection (e.g., audits of medical records) was performed to verify clinician self-reported adherence or patient attendance of follow up appointments. Future research may consider including perspectives from patient-advocates and including PCPs in the design and planning processes.

## Conclusion

This pilot study demonstrated gaps in PCP knowledge which were addressed with education. It also demonstrated improved clinician confidence in identifying, risk stratifying, and referring patients with MASLD/MASH. While some barriers relating to diagnostic access and ability to make time for care pathway interventions during a busy PCP visit persist, the authors hypothesize that many of these adoption challenges can be overcome with the integration of MASH care pathways within the EHR, streamlining the provider experience and simplifying actions required to maintain pathway adherence. Additional evidence generation is needed to demonstrate the impact of care pathway implementation on clinical care outcomes across a wider range of care delivery settings.

## Data Availability

The datasets used and/or analyzed during this study are housed on a third-party website and are available from the corresponding author on reasonable request.

## Abbreviations

AACE: American Association of Clinical Endocrinology
AASLD: American Association for the Study of Liver Disease
ADA: American Diabetes Association
AGA: American Gastroenterological Association
BMC: Boston Medical Center
CDS: Clinical decision support
EHR: Electronic health record
ELF: Enhanced Liver Fibrosis
HCC: Hepatocellular carcinoma
LFTs: Liver function tests
MASH: Metabolic dysfunction-associated steatohepatitis
MASLD: Metabolic dysfunction-associated liver disease
MRE: Magnetic resonance elastography
NAFLD: Nonalcoholic fatty liver disease
NASH: Nonalcoholic steatohepatitis
NITs: Non-invasive tests
PCPs: Primary care providers
PI: Principal investigator
VTCE: Vibration-controlled transient elastography
